# Effectiveness of mHealth consultation services for preventing postpartum depressive symptoms: a randomized clinical trial

**DOI:** 10.1186/s12916-023-02918-3

**Published:** 2023-06-26

**Authors:** Yuki Arakawa, Maho Haseda, Kosuke Inoue, Daisuke Nishioka, Shiho Kino, Daisuke Nishi, Hideki Hashimoto, Naoki Kondo

**Affiliations:** 1 Department of Health and Social Behavior, Graduate School of Medicine, The University of Tokyo, Tokyo, Japan; 2 Department of Social Epidemiology, Graduate School of Medicine and School of Public Health, Kyoto University, Kyoto, Japan; 3 Department of Medical Statistics, Research & Development Center, Osaka Medical and Pharmaceutical University, Osaka, Japan; 4 Department of Oral Health Promotion, Graduate School of Medical and Dental Sciences, Tokyo Medical and Dental University, Tokyo, Japan; 5 Department of Mental Health, Graduate School of Medicine, The University of Tokyo, Tokyo, Japan; 6 Institute for Future Initiatives, The University of Tokyo, Tokyo, Japan

**Keywords:** Postpartum depression, mHealth, Prevention, Randomized controlled trial, Health consultation services, eHealth, Mental health

## Abstract

**Background:**

Although many conventional healthcare services to prevent postpartum depression are provided face-to-face, physical and psychosocial barriers remain. These barriers may be overcome by using mobile health services (mHealth). To examine the effectiveness of mHealth professional consultation services in preventing postpartum depressive symptoms in real-world settings, we conducted this randomized controlled trial in Japan, where universal free face-to-face perinatal care is available.

**Methods:**

This study included 734 pregnant women living in Yokohama city who could communicate in Japanese, recruited at public offices and childcare support facilities. The participants were randomized to the mHealth group (intervention, *n* = 365), where they could use a free app-based mHealth consultation service with gynecologists/obstetricians, pediatricians, and midwives whenever and as many times as they wanted between 6 p.m. and 10 p.m. on weekdays throughout their pregnancy and postpartum periods (funded by the City of Yokohama government) or the usual care group (control, *n* = 369). The primary outcome was the risk of elevated postpartum depressive symptoms, defined as Edinburgh Postnatal Depression Scale score ≥ 9. Secondary outcomes were self-efficacy, loneliness, perceived barriers to healthcare access, number of clinic visits, and ambulance usage. All outcomes were collected three months post-delivery. We also conducted subgroup analyses assessing the differences in the treatment effect by sociodemographic status.

**Results:**

Most women completed all questionnaires (*n* = 639 of 734, response rate: 87%). The mean baseline age was 32.9 ± 4.2 years, and 62% were primipara. Three months post-delivery, women in the mHealth group had a lower risk of elevated postpartum depressive symptoms (47/310 [15.2%]) compared to the usual care group (75/329 [22.8%], risk ratio: 0.67 [95% confidence interval: 0.48–0.93]). Compared with the usual care group, women in the mHealth group had higher self-efficacy, less loneliness, and fewer perceived barriers to healthcare access. No differences were observed in the frequency of clinic visits or ambulance usage. Furthermore, in the subgroup analyses, we did not find differences in the treatment effect by sociodemographic status.

**Conclusions:**

Local government-funded mHealth consultation services have a preventive effect on postpartum depressive symptoms, removing physical and psychological barriers to healthcare access in real-world settings.

**Trial registration:**

UMIN-CTR identifier: UMIN000041611. Registered 31 August 2021.

**Supplementary Information:**

The online version contains supplementary material available at 10.1186/s12916-023-02918-3.

## Background

Depression during the perinatal period is a common disorder in many countries and an estimated global prevalence of postpartum depression (PPD) is 17.7% [1]. Perinatal depression can cause maternal self-harm or suicide^—^ the leading cause of perinatal death [2–4]. The prevalence of perinatal depression has been exacerbated since the outbreak of COVID-19 [5], potentially owing to reduced interpersonal communication and face-to-face care related to government recommendations for physical distancing [6]. Given that maternal depression can occur throughout the perinatal period [7], the prevention of depression from the antenatal to the postpartum period is becoming increasingly important.

Although screening and preventive interventions for depression during the perinatal period have been recommended and implemented [8–12], most women who need mental support are undetected and untreated because of several barriers [13], such as lack of knowledge [14], cost [15], insufficient available time [16], and fear of stigma [17]. In Japan, a wide range of perinatal support is available for all mothers, including free regular health checks at medical institutions during pregnancy, cost-free screening for severe depressive symptoms after delivery, local government-provided agent visits to mothers’ homes for childcare help, and universal health insurance coverage [18, 19]. Nonetheless, the prevalence of depression in Japan is estimated to range from 14.0% to 16.3% during pregnancy and from 11.6% to 15.1% up to three months post-delivery [20], suggesting the existence of unresolved access barriers to preventive care and the need for innovative approaches to overcome these barriers among perinatal women.

Using mobile wireless technologies for public health (i.e., “mHealth” [21]) can be a valuable option to reduce such barriers and has been widely implemented in some healthcare settings [22–24]. Potential benefits of mHealth consultation services include timeliness of support, access to healthcare providers without visiting, and a reduced chance of encountering stigma-related experiences during hospital visits [15, 25]. For perinatal women, these benefits may have positive impacts on increasing help-seeking behavior and actual service use, reducing loneliness and increasing self-efficacy [26, 27], resulting in the prevention of PPD [11, 13, 28]. Studies have suggested larger access barriers to healthcare among disadvantaged perinatal women [13, 29]. The benefit of these services on maternal mental health may be stronger for socioeconomically disadvantaged populations.

To the best of our knowledge, no intervention study has been conducted on the effectiveness of mHealth consultation services in preventing PPD [30, 31]. A recent review has called for a large randomized controlled trial investigating mHealth interventions among perinatal women [32]. We hypothesized that providing mHealth services offering free and unlimited opportunities for consultation with maternity and childcare professionals would have positive effects on maternal mental health by reducing the aforementioned healthcare access barriers, resolving perinatal concerns, and increasing benefits, even in settings where universal face-to-face perinatal mental care is available. As a recent review suggested, healthcare providers who are non-specialists in mental health can play an important role in improving perinatal mental health [30]. Additionally, we hypothesized that the effects of mHealth services would vary across socioeconomic groups, given the mixed prior findings [33–36]. We conducted a two-arm, parallel-group, randomized, controlled trial to examine the effectiveness of mHealth consultation services in preventing PPD and whether the effects varied across socioeconomic backgrounds.

## Methods

### Trial design and participants

We conducted a trial in Yokohama, an urban city with the largest population size (about 3.8 million) among all municipalities in Japan, where conventional free face-to-face perinatal care services for all mothers were implemented. We enrolled participants from September 1, 2020–March 7, 2021. Initially, we recruited women in the first trimester who came to a public ward office to register their pregnancy status at the Kohoku ward. In November 2020, because of the small sample size, we expanded the target population—from women in their early pregnancy to all pregnant women—and added several recruitment methods, such as announcements on the official website, recruiting in the mother preparation classes, and distributing leaflets at public childcare support facilities and local clinics. In January 2021, we expanded the recruitment field from Kohoku ward to all wards of Yokohama city. The details of the protocol are described in Additional file 1, and its changes are provided in Additional file 2 (Supplementary methods A. Protocol changes). The inclusion criteria were i) self-reported pregnancy whose expected date of delivery was until October 31, 2021; ii) living in Yokohama city; iii) able to communicate in Japanese. Because a recent review suggested the need for universal prevention strategies [12], no exclusion criteria were established, including current mental health status. All recruited pregnant women who were interested accessed the study website, which showed the study details and participation protocol. This website explained that only participants assigned to the mHealth group could use the mHealth services for free and those assigned to the usual care group could not. Participants who agreed to participate provided their consent and their basic personal information on the website.

### Randomization and masking

Participants were randomly assigned (1:1 allocation) to either the mHealth or usual care group. We used a simple randomization computer algorithm without any options, aiming to balance the baseline characteristics. After the submission of participation agreement forms, assignment information was immediately provided to the women via the website. Owing to our study design, the participants and service providers were aware of the assignment. The data collector and analyst were blinded to the group assignments until the primary analyses were completed.

### Intervention

The intervention was mHealth consultation services provided by Kids Public, Inc. The service provider had developed and offered mHealth consultation services in other regions but never investigated the effectiveness of their services in preventing depression before this study. Participants in the mHealth group could consult healthcare professionals free of charge using their mobile device without restrictions on the frequency, from the time of assignment until four months after childbirth, funded by the City of Yokohama. The service included general consultation and emotional support related to pregnancy and childcare and did not provide formal medical diagnosis or prescription. This service was delivered through the LINE platform—the most popular mobile social network service (used by over 95% of women of childbearing age in Japan) [37]—or telephone calls. The consultants were obstetrician–gynecologists, pediatricians, and midwives with at least three years of clinical experience in maternity or childcare. The consultants provided general advice to address the participant’s health-related concerns according to the consultants’ medical specializations. All consultation procedures and general guidelines followed by consultants are described in Additional file 2 (Supplementary methods B. Intervention details). The consultants shared supportive communication skills and knowledge of preventive care for mental health problems; however, no procedures on the qualification of their skills were implemented. mHealth group participants could book available 10-min consultation times and select their preferred available consultants and methods (voice calling, text messaging/chat, and video calling) between 6 p.m. and 10 p.m. on weekdays. After childbirth, they could also have chat consultations with midwives without booking or time restrictions between 1 p.m. and 5 p.m. on Mondays, Wednesdays, and Fridays. The service provider regularly sent magazines to all users with helpful information on maternity and childcare as a push-type service, with advice to promote service use for women who needed help. The service provider had a quality control team and conducted quality assessments for consultants to improve their services.

Participants assigned to the usual care group were not provided with the mHealth consultation services. Instead, they were offered access to a website created by a research team where they could easily access information on pregnancy and childcare provided by the City of Yokohama or other public organizations, such as national hospitals.

### Outcomes measures

The primary outcome was the risk of elevated postpartum depressive symptoms, which was assessed using the Japanese version of the Edinburgh Postnatal Depression Scale (EPDS) [38, 39], a 10-item structured questionnaire widely used to screen for perinatal depression [10]. Items were scored from 0 to 3 with the total score ranging from 0 to 30, where higher scores indicated elevated depressive symptoms and a high risk of PPD. We used a validated cutoff score of above or equal to nine in the Japanese version to define elevated postpartum depressive symptoms [39]. We also compared the EPDS score as a continuous variable. The EPDS is also used to screen for elevated depressive symptoms during pregnancy, with a validated cutoff score of above or equal to 13 in Japan [40]. We also used a cut-off score of above or equal to nine to assess elevated depressive symptoms during pregnancy, referring to a previous review [20].

The secondary outcomes were self-efficacy, loneliness, perceived barriers to healthcare access, and the use of medical facilities. Self-efficacy was assessed using a parenting self-efficacy scale for mothers with infants [41]. This scale consists of 13 items with response options ranging from 1 (*I do not think so*) to 5 (*I think so*). The total score ranged from 13 to 65, with higher scores indicating higher self-efficacy. Reliability and validity have been well evaluated for Japanese mothers [41]. Loneliness was assessed using the Japanese 3-item version of the University of California, Los Angeles Loneliness Scale version 3 [42]. This scale consists of three items, with four choices per item: 1) *never*, 2) *rarely*, 3) *sometimes*, and 4) *always*. The total score ranged from 4 to 12, with higher scores indicating increased feelings of loneliness. To assess perceived barriers to healthcare access, we asked the participants if they had stopped consulting with healthcare providers despite their needs during the study period. We also asked about the extent of eight possible barriers (e.g., reporting that “medical facilities are too far”) modified from the perceived barriers questionnaire that may relate to stopping consultation [43]. Four choices were listed for each reason: *never* (0 points), *rarely* (1 point), *sometimes* (2 points), and *often* (3 points). The total score ranged from 0 to 24, with high scores indicating women perceiving more considerable barriers. The score for women with no experience of stopping consultations was zero. The use of medical facilities was assessed based on participants’ self-report, including the counts of daytime medical facility visits, in addition to regular medical checks, night/holiday visits, ambulance use for their infants, and new clinic visits for mothers’ mental health. The details of all outcome questionnaires are provided in Additional File 2 (Supplementary methods C. Outcomes measures). In addition to outcomes pre-determined in the protocol, we assessed the risk of premature birth (gestational weeks < 37 weeks) and low birth weight (birth weight < 2500 g) as offspring outcomes.

We collected data using a research website and a web-based self-report questionnaire made using Google Forms at baseline and three months after childbirth. We collected personal information, including email address, age, parity, and family structure as baseline data when the participants enrolled on the research website. After the assignment, the research team sent an email to the participants asking them to answer an online questionnaire to collect additional baseline data, including sociodemographic data (trimester, household income, and education level), EPDS, and any past mental health problems. We confirmed childbirth days using birth registrations from the City of Yokohama. If we could not confirm the childbirth day from the public registration, we directly asked the participants about their child’s birthday by email. Three months after childbirth, we sent the participants an email, asking them to answer a second online questionnaire to assess the outcomes. Participants assigned to the usual care group received 500 JPY (approximately four USD) vouchers when they answered the baseline questionnaire after the assignment. All participants received 1,500 JPY (approximately 11 USD) vouchers when they answered the questionnaire three months after childbirth.

### Statistical analysis

The target sample size was 720 pregnant women, with 360 in each group, to detect an absolute difference of 7% in the risk of elevated postpartum depressive symptoms three months after childbirth between the groups. This calculation was based on the preliminary data of mothers using mHealth services in Yokohama city (proportion of EPDS ≥ 9 before and after using mHealth services; 11% and 4%, respectively), with 80% power and 5% two-sided significance level, assuming 30% attrition rate referring to other internet-based research in Japan [44, 45]. Analyses were conducted according to the intention-to-treat principle. For the primary and secondary outcomes, Fisher’s exact tests were performed for binary variables, and t-tests and Mann–Whitney U tests were performed for continuous variables to compare the outcomes three months after childbirth between the groups with complete-case analyses. We used modified Poisson and multiple linear regression analyses, as appropriate, to obtain risk ratios and 95% confidence intervals (CIs) for the primary and secondary outcomes [46].

To examine the heterogeneity in the intervention effects across sociodemographic backgrounds, we conducted subgroup analyses by age (< 35 years or older), parity (primipara or multipara), income (tertile among participants), education (< 16 years or longer), elevated depressive symptoms at participation (EPDS score < 13 or ≥ 13), and past mental health problems. As we expanded the participants from those in the first trimester to all those pregnant, we conducted an additional subgroup analysis of trimesters (first trimester vs. second and third trimesters) besides the prespecified subgroups.

As a sensitivity analysis, we conducted an all-case analysis with multiple imputations to handle missing data, using the same model as the primary analysis [47]. Because some participants were included early in their pregnancy, some missing data due to miscarriage could not be avoided. The missing values for baseline characteristics and the primary outcome were imputed using a sequence of independent univariate conditional imputation methods because the missing values showed a monotone pattern. The model included random assignment indicators, age, parity, household size and calculated days to birth at participation to impute missing variables from other baseline variables and EPDS scores three months post-delivery. We generated 20 simulated data sets, and the results were combined using Rubin's rule to obtain the treatment effect estimate and 95% confidence intervals.

To estimate the complier-average causal effect of mHealth use, we compared women according to their actual usage of mHealth based on consultation records using random allocation as an instrumental variable, while accounting for potential selection biases after randomization [48]. To explore the components of the mHealth service that contributed to the effects, we compared the primary outcome according to the number of consultations (once, 2–4 times, ≥ 5 times), specialties of each healthcare consultant, and methods of each consultation type (voice calling, text chat messaging, and video calling) with adjustment of baseline characteristics including age, parity, trimester, household size, income, education, elevated depressive symptoms at baseline, and past mental health problems. All analyses were performed using Stata software, version 16 (StataCorp), conducted by the first author and the second author, who was Certificated Senior Epidemiologist by the Japan Epidemiological Association.

### Project information and funding

This trial was executed under the model project of Social Impact Bond—one of the styles of Pay for Success policy constructed by the City of Yokohama—with a business contract between the City of Yokohama, Kids Public, Inc., the University of Tokyo, and other stakeholders. Kids Public, Inc. collected personal information, such as names and e-mail addresses, as part of their services and shared this data with the research team under the participants’ agreement. The research team was solely responsible for constructing the questionnaire, collecting baseline and outcome data, conducting the analyses, and drafting the manuscript. Details of the Pay for Success project are described in Additional file 2. The study protocol was approved by the ethical review board of the University of Tokyo (no. 2019347NI).

## Results

**Fig. 1:**
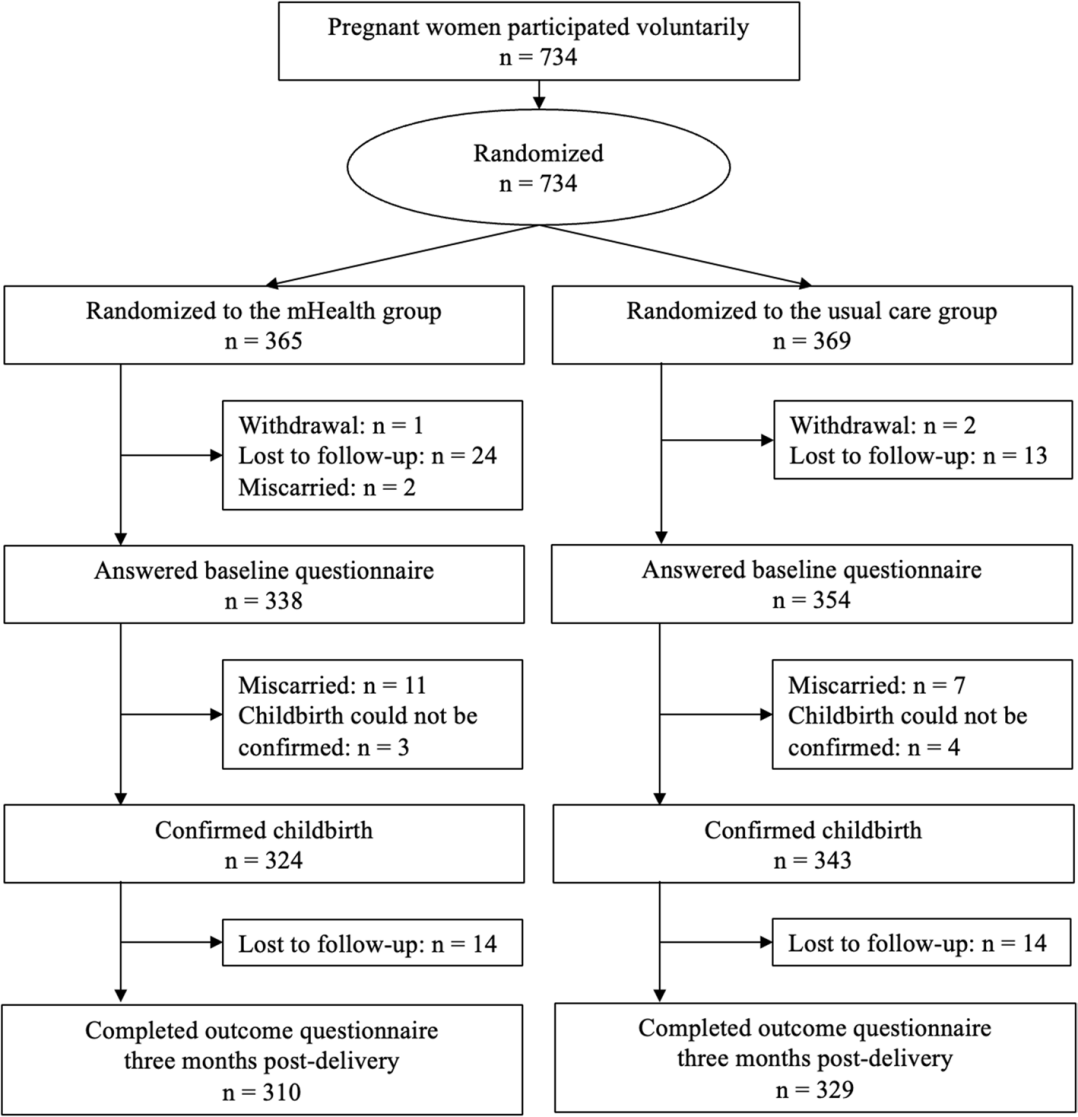
CONSORT Diagram

A total of 734 women participated. All women were randomly assigned to either the mHealth (*n* = 365) or usual care (*n* = 369) groups. We confirmed 324 and 341 deliveries from the mHealth and usual care groups, respectively. After randomization, three women withdrew their participation, and 20 women miscarried. Finally, 310 and 329 women, with a mean age of 32.9 ± 4.1, completed the outcome questionnaire in both groups, respectively (response rate: 87%; attrition rate: mHealth group, 15.1%; usual care group, 10.8%; see Fig. 1). The demographics and baseline characteristics were similar between groups (Table 1). At baseline, 5.2% of the mHealth group and 7.9% of the usual care group had elevated depressive symptoms using EPDS ≥ 13, and 20.7% of the mHealth group and 21.9% of the usual care group using EPDS ≥ 9. Comparisons of participants who were lost to follow-up and those who completed the study are illustrated in Additional file 2: Table S1.

**Table 1:** Participants’ baseline characteristics

Characteristics	mHealth group (*n* = 310)	Usual care group (*n* = 329)
Maternal age, years		
Distribution, no. (%)		
20–29	67 (21.6)	67 (20.4)
30–34	138 (44.5)	150 (45.6)
35–45	105 (33.9)	112 (34.0)
Mean [SD]	32.8 [4.1]	33.0 [4.2]
Married or partnered, no. (%)	309 (99.7)	329 (100)
Primipara, no. (%)	196 (63.2)	198 (60.2)
Gestational age at participation, no. (%)		
First trimester (≤ 13 weeks)	129 (41.6)	143 (43.5)
Second trimester (14–27 weeks)	62 (20.0)	71 (21.6)
Third trimester (≥ 28 weeks)	119 (38.4)	115 (35.0)
Household size, no. (%)		
1	2 (0.7)	1 (0.3)
2	188 (60.7)	190 (57.8)
≥ 3	120 (38.7)	138 (42.0)
Equivalent household income, no. (%)		
Low (≤ 2.5 million yen)	102 (32.9)	101 (30.7)
Intermediate (> 2.5 & ≤ 4.5 million yen)	159 (51.3)	159 (48.3)
High (> 4.5 million yen)	48 (15.5)	68 (20.7)
Unknown	1 (0.3)	1 (0.3)
Education, no. (%)		
< 16 years (Under university)	77 (24.8)	71 (21.6)
≥ 16 years (University or higher)	233 (75.2)	258 (78.4)
Elevated depressive symptoms at baseline, no. (%)		
EPDS ≥ 13	16 (5.2)	26 (7.9)
EPDS ≥ 9	64 (20.7)	72 (21.9)
Having past mental health problems, no. (%)	20 (6.5)	35 (10.6)

*Abbreviations*: *EPDS* Edinburgh Postnatal Depression Scalem, *SD* standard deviation

**Fig. 2:**
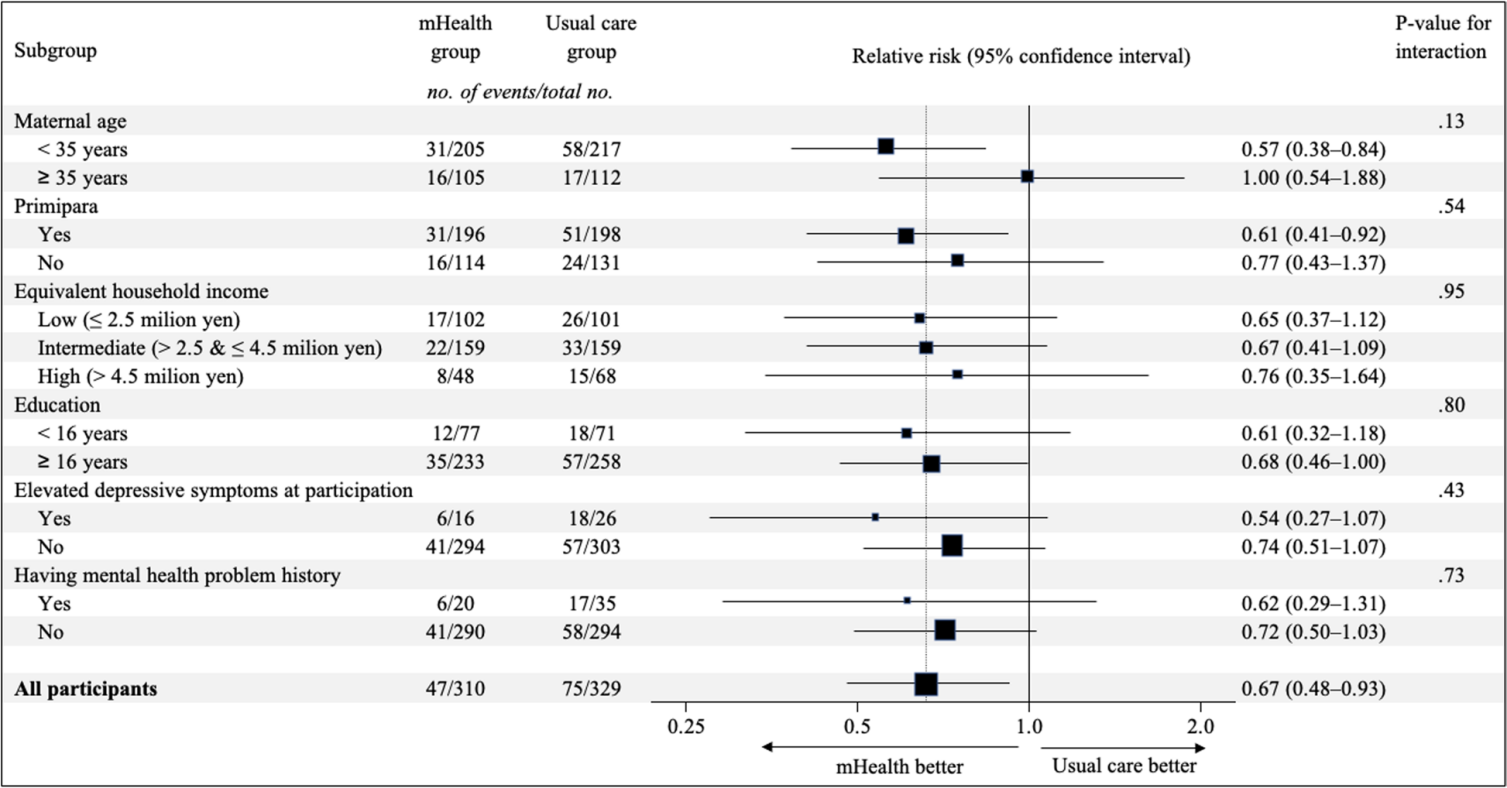
Subgroup analysis: relative risks of elevated postpartum depressive symptoms per participants’ characteristics. Abbreviation: EPDS, Edinburgh Postnatal Depression Scale

Women assigned to the mHealth group had a lower risk of elevated postpartum depressive symptoms (47 of 310 women [15.2%]) compared to the usual care group (75 of 329 women [22.8%]; relative risk, 0.67 [95% CI, 0.48–0.93]; Table 2). The mean EPDS score was also lower in the mHealth group compared to the usual care group (4.7 vs. 5.6, mean difference: -0.84 [-1.5 to -0.18]). The results did not change in the sensitivity analysis using multiple imputations for missing data (relative risk, 0.68 [95% CI, 0.49–0.94]). Compared to the usual care group, women in the mHealth group displayed higher parenting self-efficacy (47.9 vs. 46.9, mean difference: 0.97 [95% CI, -0.05 to 1.98]), lower loneliness scores (6.78 vs. 7.17, mean difference: -0.39 [95% CI, -0.73 to -0.04]), and lower perceived barriers to healthcare access (4.1 vs. 5.2, mean difference: -1.05 [95% CI, -2.12 to 0.02]). We found no difference between the groups in medical facility visits for children, ambulance usage for children, and new clinic visits for mothers’ mental health. We also found no difference between the groups in the risk of premature birth and low birth weight offspring outcomes (Additional file 2: Table S2). We did not find heterogeneity in the intervention effects among all subgroups (Fig. 2), including those by income or education level. The additional subgroup analysis also indicated that findings did not differ by the participation timing (first trimester; relative risk = 0.72 [95% CI: 0.43–1.19], second and third trimesters; relative risk = 0.63 [95% CI: 0.41–0.97], P-value for interaction = 0.71).

**Table 2:** Primary and secondary outcomes by groups

Outcome	mHealth group (*n* = 310)	Usual care group (*n* = 329)	Relative risk^a^ (95% CI)	Mean difference^b^ (95% CI)	*P* Value
**Primary outcome, no. (%) or mean [SD]**					
Elevated postpartum depressive symptoms (EPDS ≥ 9)	47/310 (15.2)	75/329 (22.8)	0.67 (0.48–0.93)	-	.02
Scores of EPDS	4.7 [3.9]	5.6 [4.5]	-	-0.84 (-1.5 to -0.18)	.01
**Secondary outcome, no. (%) or mean [SD]**					
Parenting self-efficacy	47.9 [6.2]	46.9 [6.8]	-	0.97 (-0.05 to 1.98)	.06
Loneliness	6.78 [2.2]	7.17 [2.3]	-	-0.39 (-0.73 to -0.04)	.03
Barriers to healthcare access	4.1 [6.6]	5.2 [7.1]	-	-1.05 (-2.12 to 0.02)	.05
Number of medical service usage for their children					
Clinic visits during daytime	1.2 [1.6]	1.3 [1.8]	-	-0.11 (-0.38 to 0.15)	.40
Clinic visits during night/holiday	0.09 [0.63]	0.08 [0.60]	-	0.008 (-0.09 to 0.10)	.87
Ambulance use	0.04 [0.19]	0.02 [0.16]	-	0.01 (-0.01 to 0.04)	.30
New visits to a psychiatric/psychosomatic clinic	2/310 (0.65)	9/329 (2.74)	0.24 (0.05–1.08)	-	.06

*Abbreviations*: *EPDS* Edinburgh Postnatal Depression Scale, *CI* confidence interval, *SD* standard deviation

^a^Relative risks were calculated using modified Poisson regression models

^b^Mean differences were calculated using linear regression models

Among the women assigned to the mHealth group, 224 women (72.3%) consulted with healthcare providers through mHealth once or more. The number of consultations women received ranged from 0 to 40 (median: 1, mean: 2.8). The consultation topics selected by participants before consultations widely varied, including abdominal symptoms, the fetuses, medication, breastfeeding, skin rashes affecting their children, and infection-related concerns such as COVID-19. The consultations categorized under mental health by the participants were also included, but the proportion was relatively small (2.3%, 23/1007). All topics by consultants are described in Additional file 2: Table S3. The result comparing women who used mHealth with other participants using the instrumental variable method was consistent with the primary analysis (relative risk, 0.54 [95% CI, 0.31–0.94]). In our exploratory analyses, women who consulted to 2–4 times, consulted with midwives once or more, or consulted via chat once or more tended to have low risks of elevated postpartum depressive symptoms (Table 3).

**Table 3:** Risk of elevated postpartum depressive symptoms (EPDS ≥ 9) by mHealth use status

	Using mHealth consultation	mHealth non-use or in usual care group	Relative risk^a^ (95% CI)	*P* Value
Using any type of mHealth consultation	27/224 (12.1)	95/415 (22.9)	0.57 (0.38–0.84)	.005
Total number of consultations^b^				
1	9/67 (13.4)	-	0.69 (0.36–1.31)	.26
2–4	5/89 (5.6)	-	0.27 (0.12–0.64)	.003
≥ 5	13/68 (19.1)	-	0.82 (0.48–1.42)	.48
Specialty of consultants (at least once)^c^				
Midwife	22/175 (12.6)	100/464 (21.6)	0.61 (0.40–0.94)	.02
Obstetrics–gynecologist	15/111 (13.5)	107/528 (20.3)	0.69 (0.41–1.17)	.17
Pediatrician	13/83 (15.7)	109/556 (19.6)	0.81 (0.47–1.40)	.46
Method of consultations (at least once)^d^				
Chat message consultation	22/200 (11.0)	100/439 (22.8)	0.51 (0.34–0.79)	.002
Voice call consultation	16/80 (20.0)	106/559 (19.0)	1.04 (0.66–1.66)	.86
Video call consultation	2/10 (20.0)	120/629 (19.1)	1.18 (0.32–4.37)	.81

*Abbreviations*: *EPDS* Edinburgh Postnatal Depression Scale, *CI* confidence interval

^a^Modified Poisson regression analysis with baseline adjustment was used to calculate relative risks. The adjustment variables were age, parity, trimester, household size, income, education, elevated depressive symptoms at baseline, and past mental health problems

^b^The explanatory variables were the total number of consultations, while the reference category was no mHealth consultation usage

^c^Relative risks were calculated separately for each consultant specialty. The participants were counted in each consultant category

^d^Relative risks were calculated separately for each consultation method. The participants were counted in each method of consultation category

## Discussion

Compared to the participants assigned to the usual care group, those assigned to the mHealth group—where they could use mHealth consultation services according to their needs free of charge—had a lower risk of elevated postpartum depressive symptoms three months after delivery. Women assigned to the mHealth group tended to display higher parenting self-efficacy, lower levels of loneliness, and fewer perceived barriers to healthcare access. No differences were observed in the number of medical facilities used between the groups. Among the participants in the mHealth group, those who consulted with midwives and those who used the chat consultations tended to have a decreased risk of elevated postpartum depressive symptoms. Importantly, despite the general concerns that new technology may expand the socioeconomic disparity in health and healthcare use [35, 49, 50], we did not find any evidence supporting this concern, demonstrating no differences in the potential intervention effects by income level or educational attainment.

The difference between our findings and those of a recent study indicating mixed results on the effect of mHealth-based prevention for PPD may be attributed to the features of timeliness [31]. Shorey et al. found that an mHealth app providing psychoeducation and once-a-day text-based asynchronous communication with midwives did not have a preventive effect on PPD [51]. Contrastingly, Chan et al. found a lower risk of depressive symptoms among women who could use the mHealth app, which allowed them to access useful information about pregnancy and childcare and message obstetricians with questions about these topics [52]. As these studies provided non-real-time asynchronous communication, the time intervals of their communications were unclear, and these lengths may be related to the effect differences.

Compared to these studies, the most remarkable advantage of our intervention was the timeliness and appropriateness of the consultation, as the services provided technology-based interactive, synchronous communication. Although reservations were necessary for consultations, women could consult even after a few minutes when they needed help if a professional was available. After childbirth, women could consult with midwives through text-based chatting without reservation or time limitations. A participant in the mHealth group commented in an open-ended questionnaire asking about the study participation, at three months post-delivery, that, “When I had concerns or something I did not know about childcare, I researched these things by myself, but I could not judge whether the results I found suited me or determine what I should do. At that time, it was very reassuring to be able to consult with healthcare professionals immediately through mHealth service.” (All comments related to the intervention were listed in Additional file 2: Table S4). During the consultation, women could communicate bidirectionally to ask about their concerns and clarify confusion within the service time limit, obtaining individualized advice [53]. These timely, individualized support were critical given the continuous struggles among mothers in childcaring at home [54, 55], as availability and appropriateness are essential domains in healthcare access to improve health outcomes [23, 28, 56].

Removing psychological barriers to healthcare may be another critical mechanism for preventing postpartum depressive symptoms. Using the mHealth app can be a strategy to overcome fear of stigma or judgment in vulnerable populations [13, 16, 57]. For example, women could consult on sensitive issues, such as depressive mood or low motivation to raise children, without seeing anyone or suffering emotional distress due to judgment [58, 59]. Studies suggest that text messaging is an acceptable way to consult people with mental health issues [60, 61]. In our study, chat consultation was most preferred and used, and women who consulted by chat tended to display a lower risk of elevated depressive symptoms than those who consulted using other methods. Well-designed mHealth interventions can be effective not only for privileged populations but also for resource-limited, socioeconomically vulnerable populations [33, 34, 62]. Because our user-friendly intervention was acceptable and worked well for overcoming psychological barriers for most mothers, our results might provide evidence of a preventive effect on depressive symptoms regardless of socioeconomic status [59, 63].

Other potential mechanisms include enhanced parenting self-efficacy [27, 64, 65] and reduced loneliness via the mHealth consultation. Conducting this study during the COVID-19 pandemic may have highlighted participants’ loneliness [66]. Our intervention may offer ideal connections with healthcare providers, contributing to alleviating loneliness and depressive symptoms [6, 11]. The fact that there was no difference in medical facility usage might mean that our intervention worked, not by promoting advanced psychological treatment, but by pausing the progression of depressive symptoms without additional healthcare services.

This study has several limitations. First, the generalizability of our findings is limited given that our study participants were only Japanese speakers having Internet access and almost all participants were over 20 years of age with a partner. The lack of clear standardized procedures during consultations also limits generalizability. When implementing similar services that this study evaluated, the content should be arranged according to the setting in which they are implemented. We also note that the effect size of the service shown in this study may vary depending on the setting. As our study participants had a higher percentage of elevated depressive symptoms at baseline compared to the previous review, partially due to the COVID-19 pandemic, the intervention effect may differ in situations outside the pandemic. Second, the attrition rate (13%) may lead to biased results, although sensitivity analysis with multiple imputations showed nearly the same results. Third, we did not use a formal clinical diagnosis of PPD as the primary outcome; however, the EPDS is a well-validated scale for the diagnostic interview for depression around the world [67], and is the best available patient-reported screening measure for PPD [68]. Fourth, while most of the participants were Japanese, our data did not allow us to assess the effect difference across race/ethnicity because such information was not obtained in our study. Fifth, we also could not distinguish women who had a history of mental health problems before pregnancy from those who developed depression during perinatal periods, limiting further exploration of effect heterogeneities by the type of previous mental health problems. Sixth, we did not analyze implementation costs. Performing a cost-effective analysis and comparing it with other interventions is desirable. Finally, because the details of the consultation and the provider’s answer were unknown, the components of the communication that contributed to alleviating depressive symptoms and why they worked need to be explored.

## Conclusions

This was the first randomized controlled trial to investigate the effectiveness of mHealth consultation services in preventing PPD during the COVID-19 pandemic. Our study showed the considerable potential of mHealth interventions and possible mechanisms to prevent PPD and ensure equity in services in real-world settings. Removing physical and psychosocial barriers to healthcare through mHealth can be a valuable option to improve perinatal mental health in local government policies. Future studies should explore the effective components of mHealth consultation and detailed mechanisms for preventing PPD and evaluate whether these services reduce the disparity in PPD onset across socioeconomic statuses [34, 63].

## Supplementary Information

**Additional file 1.** Study protocol.

**Additional file 2:** Protocol changes. Intervention details. Outcome measures. Details of the Pay for Success project. **Table S1.** Comparisons of participants who were lost to follow-up and those who completed the study. **Table S2.** Offspring outcomes. **Table S3.** Consultation topics by consultants. **Table S4.** Comments to the study in an open-ended questionnaire at three months post-delivery.

## Data Availability

The data are available from the corresponding author on reasonable request.

## Abbreviations

EPDS: Edinburgh Postnatal Depression Scale
PPD: Postpartum depression
