# Supplementary material for: Effectiveness of mHealth consultation services for preventing postpartum depressive symptoms: a randomized clinical trial
Source: BMC Med. 2023 Jun 26;21:221. doi: 10.1186/s12916-023-02918-3 (PMC10294407; doi:10.1186/s12916-023-02918-3)
Supplement: Supplementary file 1 — Additional file 1. Study protocol. [file 12916_2023_2918_MOESM1_ESM.docx]

Study Protocol

**１. Title**

Effects of online medical consultation services on preventing postpartum depression in pregnant women and its social impact: a randomized controlled trial

(Ethics review number: 2019347NI）

**2. Research Conducting Team**

Principal researcher: Prof. Hideki Hashimoto, Department of health and social behavior, the University of Tokyo

Co-researcher: Naoki Kondo, Department of health and social behavior, the University of Tokyo

Co-researcher: Yuki Arakawa, Department of health and social behavior, the University of Tokyo

Co-researcher: Maho Haseda, Department of Health and Social Behavior, the University of Tokyo

Co-researcher: Kosuke Inoue, Department of Social Epidemiology, Kyoto University

Co-researcher: Daisuke Nishioka, Department of Medical Statistics, Research & Development Center, Osaka Medical and Pharmaceutical University

Co-researcher: Shiho Kino, Department of Oral Health Promotion, Faculty of Density, Tokyo Medical and Dental University

Co-researcher: Daisuke Nishi, Department of Mental Health, Graduate School of Medicine, The University of Tokyo

Co-researcher: Mitsuru Nakao, the City of Yokohama

<Study periods>

The approved day of ethics review ~ 2025.03.31

<Inclusion periods>

2020.09.01 ~ 2021.10.31

**2-1. Study Object**

The impacts of postpartum depression on mothers and children are well known, and postpartum depression is related to the risks of the suicide of mothers, the delay of development of children, and the mal-social behavior of children. The previous study reported that 12.8% of all mothers were at high risk of postpartum depression. Therefore, it is not only an issue of the mother's health but a big problem with a high impact on children and their social environment. Because the continuous fear about pregnancy or delivery and the lack of social support from families or friends are risk factors for postpartum depression, it is important to provide support for mothers who need it. However, as a shortage of human and time resources in medical facilities and public agencies, it is too challenging to provide face-to-face support for all mothers with worries or questions about pregnancy and their health.

Recently, the research on online medical consultation or online treatment is expanding as support besides medical or public facilities. Online medical consultation services may reduce mothers' worries by providing the chance to consult about their questions about pregnancy and health problems of their children without visiting medical facilities, resulting in reducing risks of postpartum depression. Furthermore, solving the mother's problem with online services may have positive social impacts, such as lowering the burden on mothers and families related to visiting medical facilities.

However, it is unknown that online medical consultation services can reduce the risks of postpartum depression. This study aims to investigate whether providing online medical consultation services can reduce the risks of postpartum depression through a randomized-controlled trial and explore its social impacts.

**2-2. Methods**

(1) Research design

Prospective randomized-controlled trial

(2) Sample size

ⅰ) Numbers of study participants

Intervention group: 360

Control group：360

ⅱ)Rationale of sample size calculation

We calculated the required sample size as 249 in each group to detect differences in the proportion of women who were above a cut-off of the Edinburgh Postnatal Depression Scale: EPDS between 11% and 4% referring to the previous studies and preliminary program, with 5% two-sided significance level and 80% power. Considering the expected attrition rate as about 30%, We set the target sample size as 360 in each group.

ⅲ) Criteria

1. Inclusion criteria

Pregnant women living in Yokohama City whose expected date of delivery was before October 31, 2021.

1. Exclusion criteria

Women who don’t agree the participation.

Women who were not capable of communication in Japanese.

1. Analytic criteria (Participants who met this criteria will be excluded in the supplement analyses)

Miscarriage or stillbirth.

Baby enter the intensive care unit.

Participant get sick and hospitalize after childbirth.

Ongoing treatment of depression, bipolar disorder, or other psychiatric disease at the participation.

(3) Intervention

(1) Invasion to the participants: No

(2) Intervention to the participants: Yes

●Details of the interventions：Online medical consultation services “Syounika Online

/Sanfujinka Online”

In this study, we will provide online medical consultation services “Syounika Online/Sanfujinka Online” which the enterprise Kids Public Inc offers as an intervention. This service is delivered through the LINE platform. Women can consult with obstetrician-gynecologists, pediatricians, and midwives using this mobile service platform in the time women book by chat, call, or video call.

In addition, study participants can use “Josanshi chat Soudan,” in which women can consult with midwives about childcare or feeding by chat without booking after delivery. Furthermore, the enterprise will send the message that women can consult when they want and provide helpful information for women in childcare through the app.

These services provide only consultations; diagnosis, treatments, or intervention at the medical facilities are not included. These services are not invasive and are not competitive with existing medical or public support systems but complementary to them. If medical professionals whom women consult judge women need more aggressive support, the professionals recommend visiting medical facilities or public offices to get help.

(4) Study flow


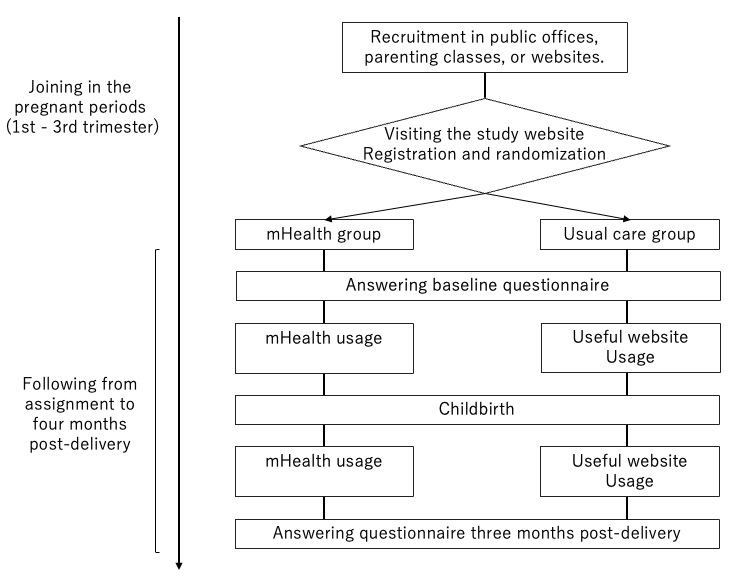
Figure１．Study flowchart.

*Online medical consultation services include “Syounika Online/Sanfujinka Online” and “Josanshi chat Soudan”.

This study is conducted under the project of Yokohama city. The member of the department of health and social behavior, the University of Tokyo, operates this study co-ordinate with Kids Public Inc., Kohoku-ward in Yokohama city, and other wards in Yokohama city.

1. Recruitment

We will recruit eligible pregnant women living in Yokohama city between September 1, 2020, and October 31, 2021, using leaflets or other media to ask them to access the study website. To inform the study information, we will distribute the leaflets with a QR code of the study website at the ward office, at the childbirth classes held or supported by Yokohama city, and at the medical facilities in Yokohama city. In addition, we will announce our study information, the QR code, and the URL of the study website on the official Twitter account, on the official website of Yokohama city, on the official newspaper of Kohoku ward, and by the official letter for pregnant women from Yokohama city. Pregnant women can access the study website through these announcements.

Figure 2. Assumed image of the leaflet.


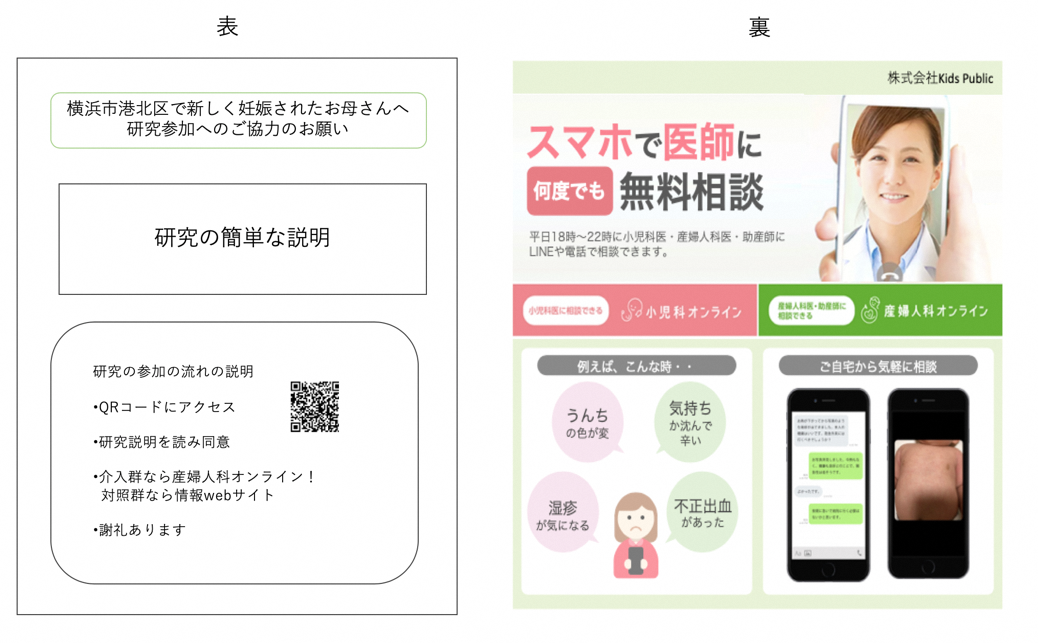


② How to obtain the agreements, get the baseline personal information, and randomize the participants.

Pregnant women will spontaneously access the study website and be checked the eligibility on the website. After that, they read and understand the study protocol details, the offered intervention, randomization, and the deal of their personal information. We will inform the women that the study team of the University of Tokyo, Yokohama city, and Kids Public Inc. will share their minimum required private information to conduct this study. Women who want to participate in this study will agree with the participation and enter their personal information, including name, birthday, mail address, mobile phone number, postal code, expected day of delivery, parity, and family structure, on the website. After sending the agreement, an online system will immediately generate the study ID and 1:1 random allocation to either the intervention group or the control group without using any personal information. Participants will know their random assignment shown on the website and by e-mail.

After allocation, participants will receive an online questionnaire sent by e-mail from the study team of the University and answer it. This questionnaire will ask about baseline participants' information, such as the primary outcome of EPDS, gestational age, loneliness, past mental health problems, income, education, and social support.

Figure 3. Assumed image of the study website.


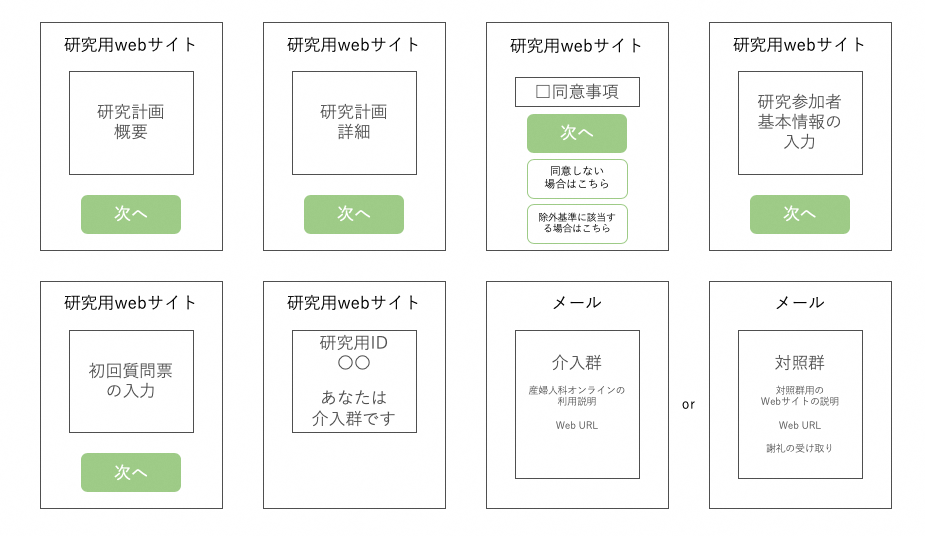


③ Details of information about the intervention group and the control group

1. The intervention group

Pregnant women in the intervention group will be provided the online consultation services, “Syounika Online/Sanfujinka Online” from the participation to four months after delivery, and the services “Josanshi chat Soudan” four months after delivery. Usually, these services need a usage fee; however, study participants can use them free without limitations on the number of times.

<Features of the intervention>

- Users can book any available time and consult anything they want for ten minutes during the service hours, which are Monday through Friday between 6 PM and 10 PM.
- Users can choose preferred methods, including voice calling, chat messaging, and video calling
- Users can also consult with midwives by chat without booking after the childbirth between 12 PM and 6 PM on the 2-3 times open weekday.
- Service provider constantly informs the users at least once a week that users can consult anything if they want through the platform.

Figure 4. The image of online consultation services (Intervention group)


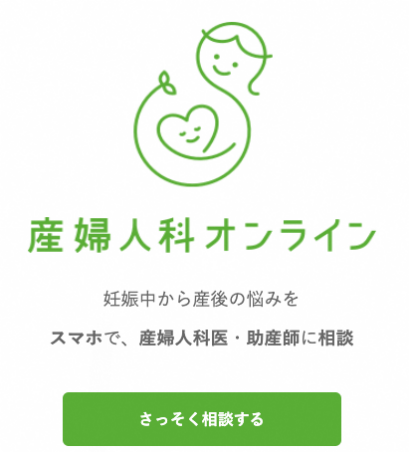
　　　
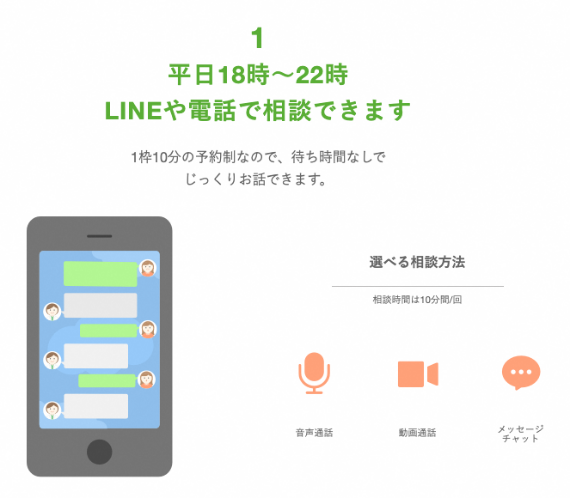


Women in the intervention group will get the online survey of EPDS two months after delivery offered by the service provider as a standard service. Women having high scores will be recommended to consult with professionals in the app.

1. The control group

Women in the control group cannot use the online consultation services above. Instead, they can use the website made by the research team of the University, which offers URLs of the useful public websites about pregnancy and childcare. Women in the control group also can get the online voucher after answering the baseline questionnaire.

Figure 5. The Image of the website offering the URLs of the useful websites (Control group)


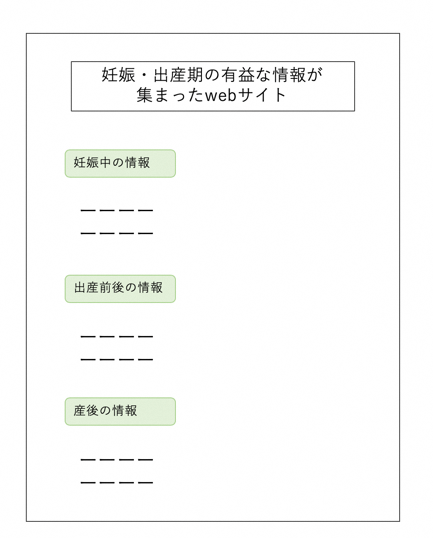


Because this is an effectiveness study to evaluate the services, which have already been introduced in several public agencies or companies, women assigned to the control group can buy the services of their own will, and we will not prohibit it.

④ Online questionnaire of the outcomes three months after delivery

The research team of the University will get the childbirth date of all participants who submit the registration of birth to the public office from the agencies of Yokohama city with the participants’ agreement.

A research member will send an online questionnaire three months after delivery by e-mail. This questionnaire includes the primary outcome of EPDS and other outcomes such as self-efficacy, loneliness, and usage of the medical facilities. Participants will get an online voucher after answering this questionnaire regardless of the assigned groups.

⑤ Analytic methods

This study aims to assess the difference in the proportion of EPDS above the cut-off three months after delivery between the intervention group and the control group. Analyses will be conducted according to the intention-to-treat principle. We will conduct Fisher's exact test for the primary outcome. We will use a t-test or Mann-Whitney U test for the secondary outcomes according to the variable characteristics. The outcomes are bellow.

●Primary outcome

Proportion of the participants with the Edinburgh Postnatal Depression Scale (EPDS) above the cut-off three months after delivery（Cut-off points: nine or above）.

●Secondary outcomes

• Barriers to healthcare access

• Parenting self-efficacy

• Loneliness (the 3-items version of UCLA loneliness scale)

• Usage of the medical facilities besides regular health check

- Daytime visit

- Night, weekend, and emergency visit (outside of the business hours)

- Ambulance usage

• Participant rate to the childcare class

• Any other information needed by Yokohama city

We will conduct subgroup analyses. Subgroups are below.

●Subgroups

• EPSD above or under the cut-off at the participation

• Primipara or multipara

• Income

• Education

• Age (under 18 years old or above)

• With or without partner

• Having past mental health problem or others

**3. Burdens, expected risks, and benefits for study participants.**

(1) Physical and psychological burdens and potential risks.

• Participants don't need any charges for online consultation services except for internet usage or voice call charges.

• Participants need to answer online questionnaires two times, at the participation and three months after delivery. It will take 10 - 15 minutes.

• Personal information will be managed on the server of the service provider strictly. However, there may be psychological burdens that participants feel anxious about the leakage of their information.

(2) Benefits of participants

• All participants will get about ten dollars online vouchers of Amazon inc. when they answer the online questionnaire three months after delivery.

• Participants assigned to the intervention group can use the online medical consultation services and get health information without charge.

• Participants assigned to the control group will get about four dollars online vouchers of Amazon Inc. when they answer the online questionnaire at the time of participation.

(3) Measures to minimize the risks for the participants

• To reduce participants' psychological burden, we will announce that their personal information will be stored with a password lock and used only to collect information related to the research, evaluate social impact, and deliver online medical consultation services.

• Furthermore, we also announce that personal information will be used only by the research team members of the University of Tokyo, public agents of Yokohama city in charge of this study, and the person of Kids Public Inc. in charge of this study.

**4. Informed consent**

We clearly explain the study aim, study flow, randomization, and interventions on the study website. We also state that we will adhere to the "Ethical Guidelines for Medical and Health Research Involving Human Subjects" and "Declaration of Helsinki" on the website. In addition, we announce that participants can decide whether they participate or not of their own will, that they never have a disadvantage by not participating, and that they can withdraw the participation whenever they want.

We consider women to agree to participate in our study by checking the agreement of participation and entering their names, birthday, and mail addresses on the study website. We state the contact mail address on the study website, and we announce participants to send a mail if they want to withdraw their participation.

**5. How to deal with the personal information of the study participants.**

We obey the "Act on the Protection of Personal Information," and we carefully treat the information to protect participants' privacy.

We announce that personal information will be used only by the research team members of the University of Tokyo, public agents of Yokohama city in charge of this study, and the person of Kids Public Inc. in charge of this study. We also announce that their personal information will be used only to deliver online medical consultation services, to conduct this research, and to evaluate the social impact and that we never use other aims nor share other people who do not relate to this study.

As a general rule, during the study periods written in the protocol made by the research member of the University of Tokyo, Yokohama city will agree to conduct the primary and secondary analyses of the anonymous data in this study and to publish Japanese/English articles on the study results after checking the contents to increase the social value of this study.

(1) How to obtain the information of the participants

The information participants enter on the website will be stored on the server of the Google Cloud Platform managed by Kids Public Inc. with encryption and password rock. A research member of the University of Tokyo will get the data of name, birthday, study ID, and mail address with the password needed to conduct the research from a member of Kids Public Inc.

A research member of the University of Tokyo will obtain the baseline and outcome data from the participants by Google forms sent by email to the participants, store in the researcher's Google Drive temporarily, and be managed in the local storage in the data managing room of the University of Tokyo.

After the intervention study period, a member of Kids Public Inc. will provide all anonymous data that participants will enter at the participation to the University of Tokyo research member with a password rock. Finally, a data manager will merge all data using the study ID. An analyst of the research member will conduct the primary and secondary analyses using this data.

All data managed in a data managing room of the University of Tokyo will be stored in the local storage with a password lock and kept in a locker locked by a physical key.

(2) How to deal with the data after the study period

After the study period, all information stored on the server of Kids Public Inc. will be discarded. The data managed at the University of Tokyo will be processed in an anonymous state and kept for a long time to conduct further studies evaluating the social impacts if the participants agree.

**6. Monitoring and audit**

Monitoring and audit are not planned.

**7. Report to the research manager of the institute**

According to the procedure document, we will report our study progress to the institute's research manager using the research ethics online system.

**8. Fund and conflict of interest**

This research will be conducted under Yokohama city's "Model project of Social Impact Bond." The graduate school of medicine, the University of Tokyo, will conduct this research as contract research from Yokohama city. The University of Tokyo is funded by Yokohama city to conduct this research.

In addition, the University of Tokyo is provided free online medical consultation services, “Syounika Online/Sanfujinka Online” and “Josanshi chat Soudan,” to conduct this research from Kids Public Inc., which is contracted with Yokohama city.

As for conflict of interests, principal researcher Hideki Hashimoto, co-researcher Naoki Kondo, and co-researcher Yuki Arakawa do not get any reward or have no equities from Kids Public Inc. or Yokohama city.

**9. Contact information**

We announce the contact information below, and we will deal with questions and inquiries from participants or other people.

The Department of health and Social Behavior, School of Social Medicine, the University of Tokyo

Contact person：Yuki Arakawa

Address：Postal code 113-0033, 7-3-1, Hongo, Bukyo-ku, Tokyo

e-mail：[utokyo.research736901@gmail.com](mailto:utokyo.research736901@gmail.com)

Version 1.0 2020.03.18

Version 2.0 2020.08.30

Version 3.0 2020.10.21

Version 4.0 2021.01.15

**Summary of changes to the protocol**

Version 1.0 and 2.0 were approved before starting the recruitment.

**•Version 1.0**

The first version of the study protocol was approved by the ethical review board of the University of Tokyo on March 16, 2020.

**•Version 2.0 (approved on August 30, 2020)**

The following changes were made to the protocol.

1. The condition "women in early pregnancy (first trimester)" was added to the inclusion criteria.
2. The recruitment period was changed from between July 1, 2020, and November 30, 2020, to between September 1, 2020, and January 31, 2021, because of the operation change of the founder and the service provider.
3. The timing to answer the baseline questionnaire, except for age, parity, household size, and expected birthday was changed from before to after randomization because of operational difficulties.

We registered our trial to the UMIN-CTR Clinical Trial (identifier: UMIN000041611) on August 31, 2021.

Version 3.0 and 4.0 were approved after starting the recruitment.

**•Version 3.0 (approved on October 21, 2020)**

The following changes were made to the protocol.

1. The inclusion criteria were changed from "women in early pregnancy" to "all pregnant women whose expected date of delivery was until October 31, 2021," due to the small sample size.
2. The recruitment period was expanded until August 31, 2021.
3. We added several recruitment methods such as announcements on the website and official SNS of Yokohama city, recruitment articles in ward newsletters, recruitment in the mother preparation class or childcare support site, and placement of leaflets at obstetric clinics or hospitals.
4. The details of secondary outcomes were added.

**•Version 4.0 (approved on January 15, 2021)**

The following changes were made to the protocol.

The recruitment field was expanded from Kohoku ward to all wards in Yokohama city.

The wording of descriptions was changed to reflect the abovementioned protocol changes.

**Statistical analysis plan**

The details of the descriptions of the statistical analysis plan in the study protocol are as below.

•Estimating confidence intervals

We planned to conduct modified Poisson regression models to estimate confidence intervals for binary outcomes and linear regression models for continuous outcomes.

•Conducting subgroup analyses

We planned to perform subgroup analyses using the same primary analysis model with the interaction term of intervention and pre-specified subgroups. Our pre-specified subgroups include age (< 18 years or older), parity (primipara or multipara), income (tertile among participants), education (< 18 years or longer), having elevated depressive symptoms at participation (Edinburgh Postnatal Depression Scale: EPDS < 13 or ≥ 13), past mental health problems and partnership (with or without a partner).

After enrolment, no woman younger than 18 was in our population, and only one participant did not have a partner. Therefore, we conducted a subgroup analysis using different age cut-off points: < 35 years or older, which was the definition of delayed childbearing by the Japan Society of Obstetrics and Gynecology. In addition, we did not conduct a subgroup analysis of partnerships because of the small sample size.
