# Supplementary material for: Effectiveness of mHealth consultation services for preventing postpartum depressive symptoms: a randomized clinical trial
Source: BMC Med. 2023 Jun 26;21:221. doi: 10.1186/s12916-023-02918-3 (PMC10294407; doi:10.1186/s12916-023-02918-3)
Supplement: Supplementary file 2 — Additional file 2: Protocol changes. Intervention details. Outcome measures. Details of the Pay for Success project. Table S1. Comparisons of participants who were lost to follow-up and those who completed the study. Table S2. Offspring outcomes. Table S3. Consultation topics by consultants. Table S4. Comments to the study in an open-ended questionnaire at three months post-delivery. [file 12916_2023_2918_MOESM2_ESM.docx]

**Additional File 2**

**Effectiveness of mHealth consultation services for preventing postpartum depressive symptoms: A randomized clinical trial**

**SUPPLEMENTARY MATERIALS**

**Supplementary methods**

A. Protocol changes

B. Intervention details

C. Outcomes measures

D. Safty protocol

**Details of the Pay for Success project**

**Table S1.** Comparisons of participants who were lost to follow-up and those who completed the study

**Table S2.** Offspring outcomes.

**Table S3.** Consultation topics by consultants.

**Table S4.** Comments to the study in an open-ended questionnaire at three months post-delivery (selected only mentioned for interventions).

**Supplementary methods**

**A. Protocol changes**

At the beginning of our research in September 2020, we recruited pregnant women in the early stages of pregnancy who came to register their pregnant status at the office of Kohoku ward, which has the most childbirths in Yokohama. As almost all pregnant women come to the ward office where they live in Japan, we considered this timing to be the best to recruit the candidates.

In November 2020, owing to the small number of participants, we expanded the target population from women in early pregnancy to all pregnant women. To reach all pregnant women, we added several recruitment methods, such as announcements on the website and official SNS of the City of Yokohama, recruitment articles in ward newsletters, recruitment in mother preparation classes or childcare support sites, and placement of leaflets at obstetric clinics or hospitals. In January 2021, we expanded the recruitment field from only Kohoku ward to all wards of Yokohama city because the recruitment period was limited.

As a result, the final recruitment period was from September 1, 2020 to March 7, 2021. We followed the participants until February 2, 2022. All protocol changes were approved by an ethical review board at the University of Tokyo (no. 2019347NI).

**B. Intervention details**

The consultants included 43 obstetrician–gynecologists, 99 pediatricians, and 35 midwives (confirmed in March 2022). One obstetrician-gynecologist and two midwives among the consultants had experienced training in psychological support, and the midwives shared their knowledge with other consultants. However, other consultants had never received official training in the field of psychological treatment besides their clinical experience in hospitals or clinics.

*Consultation guide*

All consultants followed the consultation guide created by the service provider. Before starting an initial consultation, all consultants read these guidelines with the service provider’s management team. This guide consists of several sections, including policy, consultation procedure, and tips. Below are the summaries of these sections.

*(1) Policy*

The consultants should attend work remembering the points listed below.

- We contribute to society by providing our service as a supplement to the existing medical systems to meet unmet needs.

- The online medical consultation service differs from standard clinic/hospital outpatient services.

- The service is a private service, not under the national insurance medical service.

The consultants should also follow the essential points listed below.

- Consultants provide general advice for clients when they address the client’s needs.

- Consultants must avoid telling specific possible diagnoses and are unable to prescribe medicine.

*(2) Consultation procedure*

The online consultation follows the process below.

1. The consultant checks the preliminary medical questionnaire answered by the client.

2. The consultant connects to the client by online tools selected by the client (voice call, text message, or video call).

3. The consultant introduces themselves and confirms the name of the client.

4. The consultant begins with a closed-ended question about the topic that the client selected in the preliminary medical questionnaire.

5. The consultant addresses the client’s problems and concerns based on their medical knowledge and specialization.

6. The consultant finishes the consultation after checking that the client’s issues have been appropriately addressed within the time limits for nighttime consultation (10 minutes) or within service hours for daytime consultation (from 1 p.m. to 5 p.m.).

*(3) Tips*

To use the limited consultation time productively, the service provider shared tips for consultation with the consultants as follows.

- It is useful to confirm what the client’s needs are at the start of consultations.

- Identifying the problems from the offset will make communication smoother.

- If the needs are not clear, directly asking about the primary problem is a better way to identify it within a short timeframe than asking open-ended questions about concerns.

- It is good practice to convey to the clients that they can feel free to consult whenever they want without hesitation.

- If the consultants think the clients are not in a good mental state, the consultants should repeatedly tell them that it is important to express their feelings directly to someone rather than keep their feelings to themselves.

*Quality Control*

The service provider conducted user satisfaction surveys and provided feedback to consultants for all users after using the service to ensure and improve the quality of the consultants. In addition, the service provider had an internal quality control team consisting of obstetrician–gynecologists, pediatricians, and midwives. This team regularly assessed the consultants’ quality by reviewing consultation records and messages, and they advised consultants if there was room for improvement in the quality. Assessment points are listed below.

- Did the consultant start consultations on time?

- Did the consultant introduce themselves?

- Did the consultant reply to the message of chat consultation from clients within two minutes?

- Did the consultant use appropriate words?

- Did the consultant use phrases of empathy?

- Did the consultant avoid telling a specific diagnosis when they spoke about a possible disease?

- Did the consultant check to see if their client’s concerns had been addressed?

- Did the consultant have a final greeting?

- Was the consultation time appropriate (more than five minutes)?

- Did the consultant ask what advice the client needed?

- Did the consultant give back-channel feedback when the clients replied to questions?

**C. Outcomes measures**

***Secondary outcomes***

(1) *Self-efficacy*

Self-efficacy was assessed using a parenting self-efficacy scale for mothers of infants [41]. This scale consists of 13 items with five response options ranging from 1 (*I do not think so*) to 5 (*I think so*). The 13 items include, “I think I can handle any problems that may arise in raising my child”; “I can control my feelings”; “I can express my feelings straightforwardly”; “I can share my pleasure in raising my child with close people”; “People around me approve of my child care”; “I can rely on someone when I have trouble raising my child”; “I can seek advice from people around me in raising my child”; “I think there is something only I can do to raise my child”; “I can easily reach out to others”; “I can make companions who are raising children”; “I have time for doing something besides raising my child”; ‘I do not have the confidence to continue raising my child (reverse item),” and “I have an ideal image of raising my child.” The total scores ranged from 13 to 65, with higher scores indicating higher self-efficacy. Reliability and validity have been well evaluated for Japanese mothers.

2) *Loneliness*

Loneliness was assessed using the Japanese three-item version of the University of California, Los Angeles Loneliness Scale version 3 [42]. This scale consists of three items, with four choices per item: 1) *never*, 2) *rarely*, 3) *sometimes*, and 4) *always*. The three items are, “How often do you feel that you lack companionship?,” “How often do you feel left out?,” and “How often do you feel isolated from others?” The total scores ranged from 4 to 12, with higher scores indicating women feeling lonelier.

(3) *Barriers to healthcare access*

Barriers to healthcare access were assessed by asking participants whether they had stopped consulting with healthcare providers despite their needs during the study period. We also asked about the extent of eight possible barriers modified from the perceived barriers questionnaire that may relate to stopping consultation [43]. The eight possible barriers include “Medical facilities are too far”; “Waiting time in medical facilities is too long”; “To see healthcare providers are too expensive”; “I am so busy that I have no time to consult with healthcare professionals”; “I want to avoid crowds because of the fear of COVID-19”; “Healthcare professionals seem too busy to consult”; “Hesitate to consult with healthcare professionals because my consultation content is sensitive”; and “I think my symptoms are not so severe for consultation with professionals.” Four choices were listed for each reason: *never* (0 points), *rarely* (1 point), *sometimes* (2 points), and *often* (3 points). The total scores ranged from 0 to 24, with high scores indicating women perceive more considerable barriers. The score for women with no experience of stopping consultations was zero.

4) *Use of medical facilities*

The use of medical facilities was assessed through self-reporting. We asked four questions: “How often have you visited medical facilities in the daytime, in addition to regular health checks or vaccinations since delivery?,” “How often have you visited medical facilities for your child at night or on holidays since delivery?,” “How often have you used an ambulance for your child since delivery?,” and “Have you visited a psychiatrist or a psychological clinic/hospital for the first time since you became pregnant?”

D. Safety protocol

The question of EPDS item 10 asks respondents about thoughts of self-harm, with over 1 point on item 10 indicating that respondents may be at risk of self-harm. There were 71 and 62 women who scored over 1 point on the EPDS item 10 at the participation and three months post-delivery, respectively. As a safety protocol, the research staff proactively encouraged participants to consult with medical staff or public officers at the time of assessment if the participants were above the cut-off score on the total EPDS or over 1 point on the EPDS item 10.

**Details of the Pay for Success project**

This effectiveness trial was planned and executed under the model project of Social Impact Bond, one of the styles of Pay for Success policy aimed at preventing postpartum depression in Yokohama city.

The model project was constructed in collaboration with the research team (University of Tokyo), founder (the City of Yokohama), service provider (Kids Public Inc.) and other stakeholders. The study design was constructed by discussion with the founder and the service provider; next, the research team developed the protocol, which was approved by the ethical review at the University of Tokyo (no. 2019347NI).

The recruitment process was conducted in cooperation with staff members of the City of Yokohama. The research team independently constructed the questionnaire, collected baseline and outcome data except for personal information, conducted the analyses and drafted the manuscript.

The project “success” indicator was set in advance and judged by whether the service provider could reduce the risk of postpartum depression for pregnant women in the city. The service provider receives a monetary reward depending on the pre-specified rate corresponding to a risk reduction. The research team played the role of independent, objective judgement of “success” in this Pay for Success project and was not paid regardless of the results. The City of Yokohama supported the cost of conducting the research.

**Table S1.** Comparison of participants who lost to follow-up and completed the study

| Characteristics | | | mHealth group (n=365) | | Usual care group (n=369) | |
| --- | --- | --- | --- | --- | --- | --- |
|  |  |  | Lost to follow-up | Completed | Lost to follow-up | Completed |
| n | | | 55 | 310 | 40 | 329 |
| Maternal age, years | | |  |  |  |  |
|  | Distribution - no. (%) | |  |  |  |  |
|  | 20–29 | | 13 (23.6) | 67 (21.6) | 13 (32.5) | 67 (20.4) |
|  | 30–34 | | 30 (54.6) | 138 (44.5) | 12 (30.0) | 150 (45.6) |
|  | 35–45 | | 12 (21.8) | 105 (33.9) | 15 (37.5) | 112 (34.0) |
|  | Mean [SD] | | 32.3 [3.8] | 32.8 [4.1] | 32.3 [5.0] | 33.0 [4.2] |
| Married or Partnered - no. (%) | | | 55 (100) | 309 (99.7) | 39 (97.5) | 328 (100) |
| Primipara - no. (%) | | | 30 (54.6) | 196 (63.2) | 20 (50.0) | 198 (60.4) |
| Gestational age at participation - no. (%) | | | | |  |  |
|  | | First trimester | 17 (30.9) | 129 (41.6) | 15 (37.5) | 143 (43.6) |
|  | | Second trimester | 6 (10.9) | 53 (17.1) | 2 (5.0) | 65 (19.8) |
|  | | Third trimester | 5 (38.4) | 128 (41.3) | 8 (20.0) | 120 (36.6) |
|  | | Missing | 27 (49.1) | - | 15 (37.5) | - |
| Household number - no. (%) | | |  |  |  |  |
|  | | 1 | 0 (0) | 2 (0.7) | 1 (2.5) | 1 (0.3) |
|  | | 2 | 29 (52.7) | 188 (60.7) | 16 (40.0) | 190 (57.9) |
|  | | ≥ 3 | 26 (47.3) | 120 (38.7) | 23 (57.5) | 137 (41.8) |
| Equivalent household income - no. (%) | | | | |  |  |
|  | | Low (≤ 250 million yen) | 14 (25.5) | 102 (32.9) | 14 (35.0) | 101 (30.7) |
|  | | Intermediate (>250 & < 450 million yen) | 11 (20.0) | 159 (51.3) | 6 (15.0) | 159 (48.3) |
|  | | High (≥ 450 million yen) | 3 (5.5) | 48 (15.5) | 5 (12.5) | 68 (20.7) |
|  | | Unknown | 0 (0) | 1 (0.3) | 0 (0) | 1 (0.3) |
|  | | Missing | 27 (49.1) | - | 15 (37.5) | - |
| Education - no. (%) | | |  |  |  |  |
|  | | <16 years (Under university) | 11 (20.0) | 77 (24.8) | 10 (25.0) | 71 (21.7) |
|  | | ≥16 years (University or higher) | 17 (30.9) | 233 (75.2) | 15 (37.5) | 257 (78.4) |
|  | | Missing | 27 (49.1) | - | 15 (37.5) | - |
| Having past mental health problems - no. (%) | | | 3 (5.5) | 20 (6.5) | 2 (5.0) | 35 (10.6) |
|  | | Missing | 27 (49.1) | - | 15 (37.5) | - |
| High depressive symptoms at baseline (EPDS ≥ 13) - no. (%) | | | 3 (5.5) | 16 (5.2) | 1 (2.5) | 26 (7.9) |
|  | | Missing | 27 (49.1) | - | 15 (37.5) | - |

Abbreviation: EPDS, Edinburgh Postnatal Depression Scale; SD, standard deviation.

Table S2. Offspring outcomes

| Outcome | mHealth group | Usual care group | Relative risk ^a^  (95% CI) | P Value |
| --- | --- | --- | --- | --- |
| **Offspring outcomes, no. (%)** |  |  |  |  |
| Premature birth (gestational weeks < 37 weeks) | 14/310 (4.5) | 25/329 (7.6) | 0.59 (0.31–1.12) | .11 |
| Low birth weight (Body weight < 2500g) | 27/310 (8.7) | 28/329 (8.5) | 1.02 (0.61–1.70) | .93 |

Table S3. Consultation topics by consultants.

| Consultants | Topics ^a^ | Number |
| --- | --- | --- |
| Obstetrician–gynecologists – nighttime | | 255 |
|  | Abdominal symptoms | 44 |
|  | Fetus | 21 |
|  | Medication | 17 |
|  | Genital bleeding | 12 |
|  | Infection | 7 |
|  | Lochia | 7 |
|  | Mental health | 7 |
|  | Lifestyle | 6 |
|  | Menstruation | 6 |
|  | COVID-19 | 5 |
|  | Fever | 5 |
|  | Miscarriage | 4 |
|  | Puerperium | 2 |
|  | Others (not including mental health issue) | 112 |
|  |  |  |
| Pediatricians – nighttime | | 152 |
|  | Skin rash | 41 |
|  | Stool | 13 |
|  | Cough / Runny nose | 11 |
|  | Growth and development | 11 |
|  | Constipation | 11 |
|  | Nutrition | 7 |
|  | Injury | 6 |
|  | Vomiting | 3 |
|  | Fever | 2 |
|  | COVID-19 | 1 |
|  | Maternal mental health | 1 |
|  | Others (not including mental health issue) | 45 |
|  |  |  |
| Midwives – nighttime | | 99 |
|  | Breastfeeding | 24 |
|  | Feeding (Milk) | 23 |
|  | Lumbar / Pelvic symptoms | 15 |
|  | Body weight / Nutrition | 9 |
|  | Breast issue | 6 |
|  | Mental health | 4 |
|  | Mastitis | 3 |
|  | COVID-19 | 2 |
|  | Exercise | 2 |
|  | Others (not including mental health issue) | 11 |
|  |  |  |
| Midwives – daytime | | 501 |
|  | Childcare | 255 |
|  | Breastfeeding | 120 |
|  | Feeding (milk) | 46 |
|  | Health consultation of the mother | 41 |
|  | Breast issue | 17 |
|  | Mental health | 11 |
|  | Mastitis | 9 |
|  | Back pain | 1 |
|  | Swelling | 1 |

^a^ Topics of nighttime consultations were selected by participants before starting consultations. There were some additional categorizations from “others” to specific topics by the first author. Topics of daytime consultations were categorized by consultants (midwives).

Table S4. Comments to the study in an open-ended questionnaire at three months post-delivery (selected only mentioned for interventions).

| Participants (Age) | Comments |
| --- | --- |
| A (31) | “I was really encouraged by the online consultation services for up to 3 months.” |
| B (35) | “Thanks to this research, I had access to this online service which had been so helpful. I especially appreciated the service of consulting at home during the pandemic.” |
| C (35) | “I used the mHealth consultation service only once, which was a relief to me knowing that I had someone to consult whenever I wanted, besides my primary doctor.” |
| D (38) | “During the pandemic, when we could not see someone freely, the service really helped me mentally because I could ask about some concerns I could not resolve by myself but seemed so trivial that I hesitated to visit or tell my hospital. This pregnancy was my first, and the consultation services were encouraging for me because the consultants were very supportive of my worries and concerns specific to pregnancy and delivery.” |
| E (35) | “The environment in which I could consult about a small matter without hesitation relieved my anxiety.” |
| F (21) | “The service helped me before and after delivery. Since it was my first time delivering and I did not know about it, consulting with healthcare professionals and resolving my question relieved my anxiety about childcare.” |
| G (29) | “This pregnancy was my first, and the service consulting with healthcare professionals from my smartphone was very convenient and reassuring when I had concerns but was not sure if I should go to the hospital, or when I wanted to visit a hospital but could not. I think it was a source of comfort. In addition, the service was beneficial for me because I kept myself from going out due to COVID-19.” |
| H (39) | “I used the mHealth consultation service when I worried about my child's crying behavior and skin troubles, which diminished my concerns.” |
| I (30) | “Though I did not use the service very often, knowing that I could rely on the service if I had concerns reassured me, as I did not easily call for help from others during the pandemic.” |
| J (28) | “I am thankful for the experience of being able to consult with midwives and pediatricians through the mHealth platform.” |
| K (31) | “When I had concerns or something I did not know about childcare, I researched these things by myself, but I could not judge whether the results I found suited me or determine what I should do. At that time, it was very reassuring to be able to consult with healthcare professionals immediately through mHealth service.” |
